# A Chemometric Approach to Establish Underlying Connections between Lipid and Protein Oxidation and Instrumental Color and Texture Characteristics in Brazilian Dry-cured Loin

**DOI:** 10.3390/foods9040536

**Published:** 2020-04-24

**Authors:** Denes K. A. Rosario, Maraysa R. Furtado, Yhan S. Mutz, Bruna L. Rodrigues, Yago A. A. Bernardo, Jéssica D. Baltar, Patricia C. Bernardes, Mario Estevez, Carlos A. Conte-Junior

**Affiliations:** 1 Center for Food Analysis (NAL), Technological Development Support Laboratory (LADETEC), Avenida Horácio Macedo, 1281, Polo de Química, bloco C, Ilha do Fundão, Cidade Universitária, Rio de Janeiro, RJ 21941-598, Brazil; deneskarosario@ufrj.br (D.K.A.R.); yhan.mutz@ufrj.br (Y.S.M.); yagoaab@hotmail.com (Y.A.A.B.); 2 Food Science Program, Institute of Chemistry, Federal University of Rio de Janeiro, Av. Athos da Silveira Ramos, 149, Cidade Universitária, Rio de Janeiro, RJ 21941-909, Brazil; maraysafurtado@hotmail.com (M.R.F.); brunalrmlk@yahoo.com.br (B.L.R.); jessicadiogobaltar@gmail.com (J.D.B.); 3 National Institute of Health Quality Control, Oswaldo Cruz Foundation, Rio de Janeiro, RJ 21040-900, Brazil; 4 Department of Food Engineer, Federal University of Espirito Santo, Alto Universitário, s/n, Alegre, ES 29500-000, Brazil; patricia.bernardes@ufes.br; 5 Institute of Meat and Meat Products (IPROCAR), TECAL Research Group, University of Extremadura, 10003 Cáceres, Spain; mariovet@unex.es

**Keywords:** color quality, texture profile analysis, multivariate statistical analysis, nondestructive methods

## Abstract

This study aimed to use chemometrics to evaluate the influence of lipid and protein oxidation on the color and texture characteristics of Brazilian dry-cured loin (Socol, BDL). Upon exploration using hierarchical cluster analysis (HCA), two clusters were formed, indicating that higher water activity (a_w_) was associated with higher lipid and protein oxidation. However, this fact was associated with softening and low color quality (a*, chroma, and cured color). In a more in-depth exploration, using principal component analysis (PCA) for each cluster separately, connections between protein and lipid oxidation were found in high a_w_, as demonstrated by their statistical association. In the same way, relationships between high hardness and carbonyl contents were obtained only in high a_w_. In addition, an overall relationship (*p* < 0.05) between nondestructive measurements, such as hardness, and destructive methods (malonaldehyde and carbonyl contents) demonstrate that nondestructive techniques can be promising for further studies in the method replacement field. In this study, reasonable explanations of the connections between oxidative damage and quality traits in Socol are provided.

## 1. Introduction

Due to their low water activity (a_w_), dry-cured meats have been considered to be chemically and microbiologically stable products [1,2]. However, the macromolecular crowding of lipids and proteins with pro-oxidants due to dehydration as well as processing and storage conditions makes the product susceptible to oxidative reactions leading to the deterioration of this matrix [3,4]. In recent years, protein oxidation has become one of the most promising research topics in the food science field [5,6]. High protein oxidation levels are not desirable, as they contribute to the impaired color and texture of dry-cured products [4] and the loss of protein functionality [7]. In addition, they cause adverse implications to the consumer’s health [8,9]. However, protein oxidation in meat and meat products was neglected for decades, when compared to lipid oxidation [6,10].

Lipid oxidation leads to rancidity, color changes, and the formation of toxic compounds [3,11,12]. The effect of water in lipid oxidation using a low a_w_ model system [13] and food [14] is evident. Nevertheless, the connections between lipid and protein oxidation are a promising topic in the field of food science [15], but such knowledge in meat is scarce due to the high complexity of the meat matrix composition [8,9]. Some studies have explored these links in meat products with low a_w_ [4,16]. The connections between the oxidative damage of lipids and proteins and color and texture changes have been studied [4,17,18,19]; however, the full understanding remains to be elucidated.

The intrinsic relationship between food characteristics has been a field recently studied using multivariate statistical analysis [20,21,22]. In this way, chemometrics has been a statistical approach used to extract useful information from complex and large datasets [23]. Among the chemometric tools, the clustering method and principal component analysis are multivariate methods applied in all fields of science and food technology [23,24]. In this context, this study aimed to use chemometrics to evaluate (i) the influence of the key intrinsic factors on the physicochemical characteristics and (ii) the relationship between lipid and protein oxidation damage and instrumental texture and color in Brazilian dry-cured loin.

## 2. Materials and Methods

### 2.1. Brazilian Dry-Cured Loin Sample

Twenty-seven different samples (200 ± 5 g each sample) from three batches were obtained in the local market of Venda Nova do Imigrante city, in the mountain region of Espírito Santo state, Brazil. The three batches were produced in this region in the period of July–September 2017 (total production time of three months) with ripening conditions at ambient temperature (between 10.9–25.1 °C) and relative humidity (between 82.5%–84.9%) according to the Capixaba Institute for Research, Technical Assistance and Rural Extension, using a meteorological center for agroclimatic monitoring data [25]. The loins were derived from F1 pigs resulting from a cross between the landrace and large white breeds, fed with corn and soybean meal (main base) (according to pig producers) and slaughtered when the weight reached 95 kg. The loin pork was covered with NaCl for 24 h at 4 °C. After the excess salt was removed and 3 g/kg of a mixture (1:1) of powdered black pepper (*Piper nigrum*) and garlic (*Allium sativum*) was added, the seasoned loin was wrapped in pork peritoneum and ripened for three months. Immediately after the production, samples were vacuum-packed, purchased, and transported in sanitized boxes to the laboratory (25 °C) for analysis. All the samples were analyzed one day after the end of production. Intrinsic factors (water activity and cross-sectional area) and oxidation (protein oxidation and lipid oxidation) were measured, color was instrumentally determined, and texture profile analysis (TPA) was performed.

### 2.2. Water Activity and Cross-Sectional Area

Water activity was determined using Pawkit meter (Decagon Devices, Pullman, WA, USA) by placing 2 g of sample into the digital equipment, according to the method described by the manufacturer. The cross-sectional area was calculated using the formula: *A* = π × *r*^2^, and results were expressed in cm^2^. The samples had an approximately cylindrical shape, and the diameter was measured.

### 2.3. Lipid Oxidation

Lipid oxidation was determined by the thiobarbituric acid reactive substances (TBARS) method described by Yin et al. [26] and modified by Joseph et al. [27]. In brief, samples (5 g) were homogenized with 22.5 mL of trichloroacetic acid (11%) solution using an Ultra Turrax 18 basic (IKA, Wilmington, NC, USA) at 11,000 rpm, followed by centrifugation at 15,000× *g* for 15 minutes at 4 °C. The absorbance values at 532 nm were measured using a UV-1800 spectrophotometer (Shimadzu Corporation, Kyoto, Japan). The concentration of malonaldehyde (MDA) was obtained from a standard curve. The results were expressed as mg MDA/kg of dry-cured meat.

### 2.4. Protein Oxidation

The total protein carbonyl content was determined following the 2,4-dinitrophenylhydrazine (DNPH) derivatization assay based on the methods proposed by Oliver et al. [28] with modifications [29,30,31], using 3 g of sample. The absorbance was measured at 280 nm (protein content) and 370 nm (carbonyl content) in a UV-1800 spectrophotometer (Shimadzu Corporation, Kyoto, Japan). The results were expressed as nmol of carbonyl per mg of protein.

### 2.5. Instrumental Color

The color was measured using a spectrophotometer CM-600D (Konica Minolta Sensing Inc., Osaka, Japan) equipped with illuminant A, 8 mm aperture, and 10° standard observer [32]. The color analysis was performed by directly reading on the dry-cured meat at eight different internal points on each sample. In the CIE color scale, L* (lightness), a* (redness), b* (yellowness), chroma (color saturation/intensity) and hue (tonality) were measured. The determination of the color intensity of cured meat was obtained by the ratio between 650 and 570 nm, R(650/570) [32]. The ratio of 630 and 580 nm, R(630/580), indirectly estimates the concentration of metmyoglobin (MMb) on dry-cured meat surface and thus indicates color stability [32].

### 2.6. Texture Profile Analysis (TPA)

The texture profile analysis (TPA) was performed in Texture Analyzer TA-XT plus, Stable Micro Systems Ltd. (Surrey, England) equipped with a 50 kg load cell and 36 mm cylindrical probe (P/36). Samples of 10 × 5 × 10 mm (length ×·width × height) were used. Samples were compressed twice to 50% of their original height (*t* = 0 s, between the two compressions). The pretest, test, and post-test speeds were 1, 1, and 5 mm/s, respectively. Hardness, adhesiveness, springiness, cohesiveness, gumminess, chewiness, and resilience were calculated using the Exponent software package, version 6.1.9.1 (Stable Micro Systems, Surrey, England).

### 2.7. Statistical Analysis

To investigate the influence of intrinsic factors on the physicochemical characteristics of Brazilian dry-cured loin (BDL), MANOVA was performed. Henceforth, the hierarchical cluster analysis (HCA) was used following Ward’s method and Euclidean distance. To evaluate the relationship between lipid and protein oxidation and instrumental parameters of color and texture, principal component analysis (PCA) was performed in each cluster. For the formation of the principal components, the correlation matrix was used to determine significant variables (*p* < 0.05) for cluster discrimination. Pearson’s correlation was performed to evaluate the overall relationship between physicochemical characteristics. Shapiro–Wilk’s test was used to verify the data normality. Statistica 10 (Statsoft, Tulsa, OK, USA) software was used. The significance level used was 0.05. Data were standardized to eliminate the effect of the order of magnitude between the variables.

## 3. Results and Discussion

### 3.1. Influence of the Key Intrinsic Factors on the Physicochemical Characteristics of Brazilian Dry-Cured Loin

**Figure 1:**
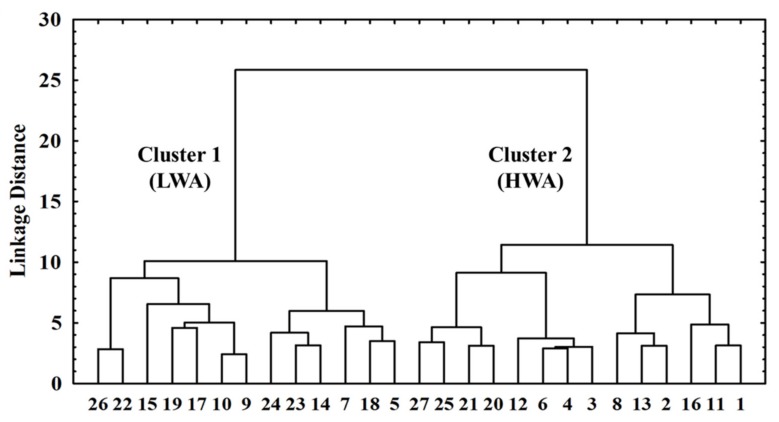
Dendrogram of Brazilian dry-cured loin samples. Cluster 1 (LWA, low water activity) and cluster 2 (HWA, high water activity).

The 27 BDL samples were separated into two clusters (Figure 1). The samples included in the cluster 1 presented lower values (*p* < 0.05) of a_w_, TBARS, and carbonyls but presented higher values (*p* < 0.05) of hardness, gumminess, chewiness, a*, b*, chroma, R(630/580), and cured color compared to the cluster 2 counterparts. Since a_w_ is a critical factor for dry-cured meat [33,34,35], cluster 1 (13 samples) was composed of samples with a low a_w_ (cluster LWA, low water activity), and cluster 2 (14 samples) was composed of samples with a high a_w_ (cluster HWA, high water activity). In this context, several observations can be made.

Several factors have been described to influence protein and lipid oxidation [9]. Among them, a_w_ plays an essential role in biochemical reactions, including redox ones [14]. As aforementioned, the values of TBARS and carbonyls were higher (*p* < 0.05) in the HWA cluster (Table 1). Water is a reaction media and may facilitate oxidative reactions by the diffusion of substrates, hence increasing the encounter of reactants [36]. For lipid oxidation, water may facilitate the attack of a radical species and the removal of hydrogen from fatty acids, thereby beginning its oxidation [36]. The a_w_ is also related to protein oxidation once the presence of water collaborates with the formation of several radicals, increasing the number of carbonyls [33,34,37]. However, dehydration leads to a molecular crowding effect that enhances the collision between lipids, proteins, and pro-oxidants, such as oxygen and iron [38]. According to the latter mechanism, loss of free water (through dehydration or even freezing) may promote oxidative reactions, yet this was not observed in the present study. Hence, apparently, there must be a particular range of a_w_ in dry-cured meat products at which the balance between the molecular crowding and molecular diffusion occurs in order for these oxidative reactions to be enhanced. For this reason, protein and lipid oxidation levels, over different a_w_ values in BDL, as well as in other dry-cured meats, should be thoroughly studied.

In addition to its involvement in the occurrence of chemical reactions, a_w_ also influences the texture and color. In this study, hardness, gumminess, and chewiness were important factors (*p* < 0.05) to discriminate against the clusters (Table 1). Low a_w_ was one of the factors strongly associated with this texture profile. Ruiz-Ramírez et al. [35] and Serra et al. [33] also found increased hardness when there was a decrease in a_w_ in dry-cured loin and dry-cured ham, respectively. Dry-cured meat products are considered foods with high hardness, gumminess, and chewiness when compared to fresh meat. In this study, higher hardness values may be related to the lower amount of water present between proteins. The water located between the proteins may decrease the direct interaction between the protein structures, hence increasing the softening [39,40]. Moreover, the cross-sectional area was not a significant variable (*p* > 0.05) for the formation of clusters (Table 1). This result indicates that standardization in the radius of the dry-cured loin allowed greater homogeneity in the water loss during the maturation/dehydration process.

As previously discussed, a_w_ influences distinct physicochemical reactions in meat products, which alter chemical, texture, and color values. In the present study, the a* value was an important variable to discriminate against the clusters (Table 1), wherein the LWA cluster obtained the highest value (*p* < 0.05). In the same way, chroma had the highest (*p* < 0.05) value in the LWA cluster (Table 1). This fact indicates a higher saturation/intensity level of the red color (a*) on LWA compared to the HWA cluster. A lower a_w_ leads to the concentration of biomolecules, including myoglobin, which, in turn, affects the color displayed by meat products. In addition to the total concentration of pigments, the chemical state of myoglobin also plays a role in the meat color. Low values of the R(630/580) ratio indicate higher concentrations of metmyoglobin [32], as presented in the HWA (Figure 1) compared to the LWA. Therefore, these data support the hypothesis that only pigment concentration does not fully explain the role of a_w_ on color quality. The higher a_w_ may have caused greater diffusion of substrates and facilitated oxidative reactions leading to discoloration [36]. High amounts of metmyoglobin cause a higher presence of brown tonality in the meat [41]. A higher amount of this oxidized pigment is a reflection of lower color stability (R(630/580)) [18,30]. Among the various functions of water in the meat, one of them is to provide greater diffusion of substances [36]. Therefore, it is possible to hypothesize that the greater diffusion/presence of oxygen caused the oxidation of Fe^2+^ into Fe^3+^, facilitating the accumulation of metmyoglobin [18] which correlates with a less intense red meat [41]. Likewise, the value of b* was also higher (*p* < 0.05) in the LWA cluster. In this study, it is possible to state that the lower oxidation due to the low a_w_ likely contributed to lesser discoloration of the dry-cured loin, resulting in the prevalence of red substances.

The values of cured color R(650/570) are presented in Table 1. According to the scale of the American Meat Science Association [32] with modifications, values ≤1.4 indicate no cured color, values of 1.5–2.1 refer to noticeable cured color, and values of 2.2–2.6 represent an excellent cured color. Although BDL presents no nitrite or nitrate addition and is an uncooked product, the values found in the present study indicate the noticeable intensity of cured color in samples classified in LWA cluster. This effect may have been due to the high concentration of substances responsible for the color resulting from the continuous and effective dehydration process. In dry-cured meat, few studies have evaluated this parameter. Böhner et al. [41] found a lower value (1.25) in cured boiled sausages when compared to the values of the present study. The lower a_w_ resulted in higher values for both R(630/580) and cured color. Therefore, we can affirm that lower a_w_ improved the overall color quality of BDL.

### 3.2. The Relationship among Physicochemical Parameters

**Figure 2:**
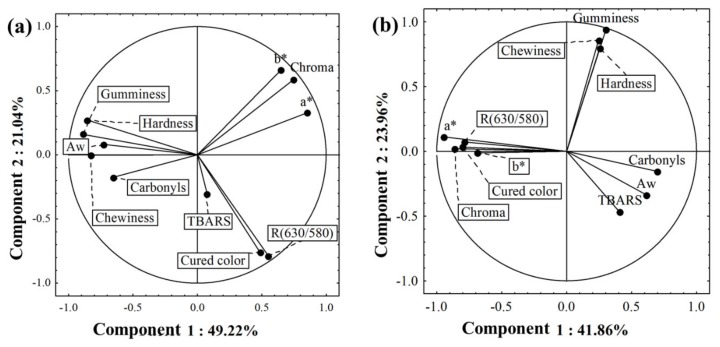
Principal component analysis of the significant variables (*p* < 0.05) in cluster 1 (LWA, low water activity) (**a**) and cluster 2 (HWA, high water activity) (**b**).

**Figure 3:**
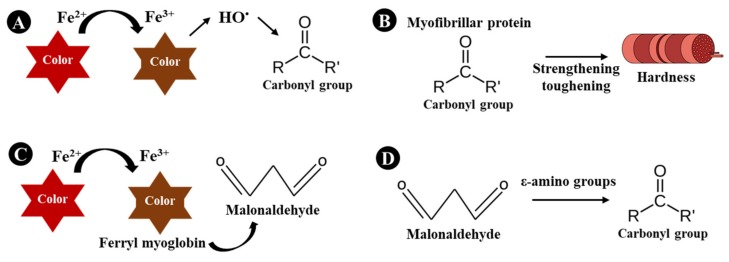
Plausible connections among physicochemical characteristics in Brazilian dry-cured loin. Connections between color and protein oxidation (**A**), texture profile and protein oxidation (**B**), color and lipid oxidation (**C**) and lipid and protein oxidation (**D**).

For a more in-depth study, principal component analysis (PCA) was performed for the LWA and HWA clusters, separately. For the LWA cluster, two components of the PCA retained 70.26% of the total variance. For the samples from the LWA, the variables a* and chroma are located in the PCA, as opposed to carbonyls (Figure 2a). These facts indicate that high a* and chroma values were associated with low protein oxidation. As previously explained, this relationship is possible due to the double role of iron, which is responsible for (i) the redness of the meat and (ii) the occurrence of oxidation, as this metal is the leading promoter of protein oxidation and discoloration reactions in meat systems [6,18] (Figure 3A).

Instrumental texture values, carbonyls, and a_w_ showed proximity in PCA (Figure 2a). This fact demonstrates that these variables are associated with each other. The low a_w_ of a meat product is mainly due to a dehydration process; as aforementioned, free water may facilitate oxidative reactions [36]. In this study, the a_w_ of 0.79 was associated with low carbonyl content and high texture values (Table 1), possibly due to low activity of meat-softening proteases. In other studies, however, dehydration caused a molecular crowding effect that increases carbonyl formation [6,38]. These effects were also described in uncured or unfermented meat products with a_w_ > 0.90. Therefore, the low level of a_w_ in the samples of cured loin was indicated as a powerfully important and possibly particular factor of this dry matrix.

For the HWA cluster, two components of the PCA retained 65.82% of the total variance (Figure 2b). High carbonyl and TBARS contents are mildly opposed to low hardness, gumminess, and chewiness values. Therefore, high oxidative damage may be associated with high hardness, gumminess, and chewiness values (due to both positive locations in PCA). Products of both lipid and protein oxidation, such as MDA and mainly protein carbonyls, are known to be involved in the formation of protein cross-links, which are believed to be responsible for the strengthening of the muscle tissue and the increase of meat toughness [6] (Figure 3B). Several studies corroborate this connection between protein oxidation and texture parameters in uncured or unfermented meat products [42,43,44]. However, in this presented study, this phenomenon only occurs in high a_w_ (0.84, HWA). In Spanish dry-cured ham with a_w_ of 0.90, using high hydrostatic pressure, a similar connection between protein oxidation and the texture was achieved [45]; however, process conditions may have influenced the connection. Therefore, a_w_ can be indicated as a key factor for all underlying connections described. However, several factors may also be associated; in this manner, the full understanding remains to be elucidated. Thiol groups, mainly cysteine, play an important role in cross-linking involving oxidation of proteins in meat [37]. Therefore, it is a field that should be considered in future studies.

For lipid oxidation, interesting differences were found between LWA and HWA clusters. In LWA, TBARS had no associations with all variables. On the other hand, in HWA, TBARS was oppositely located with color values. For this reason, high TBARS content was associated with low redness. TBARS and a* are also linked in raw meat, as myoglobin oxidation products can cause lipid oxidation [17,18] (Figure 3C). Furthermore, in the cluster HWA, the values of TBARS, carbonyls, and a_w_ showed proximity in PCA (Figure 2b). Therefore, in high a_w_ (0.84), high TBARS and carbonyl contents were associated. Cross-links of protein and lipid oxidation may occur due to the presence of malonaldehyde [15,46,47]. The formation of carbonyls (aldehydes and ketones) in proteins may be due to the covalent binding to nonprotein carbonyl compounds, such as malonaldehyde [6,15] (Figure 3D). On the other hand, in BDL with low a_w_, this phenomenon did not occur. Therefore, the a_w_ presents itself as one of the main factors necessary to fully understand in the connections between different oxidative damages in the dry matrix.

Overall correlation (Table S1) between malonaldehyde and carbonyl contents had a high/moderate-significance correlation with a* and hardness. In this way, it is possible to suggest that instrumental measurements such as color and texture have potential as indicators of lipid and protein oxidation. It is important to replace time-consuming, destructive, and environmentally unfriendly methods such as malonaldehyde and carbonyl quantification with nondestructive, fast, and environmentally friendly methods such as color and texture. Hence, we suggest further and broader studies in this field.

## 4. Conclusions

The a_w_ plays a major role in the physicochemical characteristics of BDL. In a general exploration using HCA, it was found that lower a_w_ was related to improved color quality (a*, b*, chroma, R(630/580), and cured color) and indicated lower carbonyl and malonaldehyde contents. Regarding the aspects of texture profile, low a_w_ was one of the factors that indicated high hardness, gumminess, and chewiness. In addition, it is possible to suggest that a* and hardness can be further studied as indicators of malonaldehyde and carbonyl content. In a more in-depth study using PCA, the level of a_w_ in the BDL was powerfully relevant. Connections between protein and lipid oxidation were found in high a_w_ but not in low a_w_. In the same way, the connections between high hardness and carbonyl content occurred only in high a_w_. Lipid oxidation only showed meaningful connections with instrumental color in high a_w_. In this preliminary study, information has been provided regarding BDL characteristics and their connections with oxidative damage to lipids and proteins and changes in color and texture characteristics, which will encourage further studies to improve the knowledge of the BDL and physicochemical connections in dry-cured meat.

## Figures and Tables

**Table 1:** Means and standard deviation of physicochemical characteristics obtained from the two clusters formed of Brazilian dry-cured loin.

Variables	Cluster 1 (LWA)	Cluster 2 (HWA)	*p* Values
Cross-sectional area (cm^2^)	21.69 ± 2.11	23.30 ± 2.93	0.156
Water activity	0.79 ± 0.01	0.84 ± 0.01	0.001
TBARS ^A^	0.16 ± 0.04	0.27 ± 0.07	<0.001
Carbonyls ^B^	1.93 ± 0.31	3.12 ± 0.91	<0.001
Hardness (N)	6.45 ± 0.88	5.13 ± 0.84	<0.001
Adhesiveness (g·mm)	−9.19 ± 5.71	−10.47 ± 4.60	0.543
Springiness (mm)	0.70 ± 0.05	0.71 ± 0.06	0.701
Cohesiveness	0.64 ± 0.03	0.64 ± 0.04	0.892
Gumminess (N·mm)	4099.5 ± 599.5	3286.0 ± 492.3	0.001
Chewiness (g·mm)	2898.1 ± 506.9	2335.8 ± 402.1	0.004
Resilience	0.22 ± 0.03	0.21 ± 0.02	0.304
L*	34.31 ± 2.24	33.09 ± 1.88	0.132
a*	6.62 ± 0.41	5.10 ± 0.46	<0.001
b*	6.53 ± 0.83	5.43 ± 0.71	0.001
Chroma	9.33 ± 0.82	7.50 ± 0.77	<0.001
Hue	44.68 ± 2.22	46.46 ± 2.82	0.078
R(630/580)	1.51 ± 0.02	1.41 ± 0.05	<0.001
Cured color	1.58 ± 0.03	1.44 ± 0.06	<0.001

LWA: low water activity; HWA: high water activity; ^A^: expressed as mg MDA/kg of dry-cured loin; ^B^: expressed as nmol of carbonyl per mg of protein. Results are expressed as means ± standard deviation (*n* = 27).

## Supplementary Materials

The following are available online at https://www.mdpi.com/2304-8158/9/4/536/s1, Table S1: Overall correlation between significative physicochemical characteristics (cluster analysis) on Brazilian dry-cured loin.

## References

[1] Marušić N., Petrović M., Vidaček S., Petrak T., Medić H. (2011). Characterization of traditional Istrian dry-cured ham by means of physical and chemical analyses and volatile compounds. Meat Sci..

[2] Sebranek J.G., Bacus J.N. (2007). Cured meat products without direct addition of nitrate or nitrite: What are the issues?. Meat Sci..

[3] Gandemer G. (2002). Lipids in muscles and adipose tissues, changes during processing and sensory properties of meat products. Meat Sci..

[4] Ventanas S., Estevez M., Tejeda J.F., Ruiz J. (2006). Protein and lipid oxidation in *Longissimus dorsi* and dry cured loin from Iberian pigs as affected by crossbreeding and diet. Meat Sci..

[5] Cunha L.C.M., Monteiro M.L.G., Costa-Lima B.R.C., Guedes-Oliveira J.M., Alves V.H.M., Almeida A.L., Tonon R.V., Rosenthal A., Conte-Junior C.A. (2018). Effect of microencapsulated extract of pitaya (*Hylocereus costaricensis*) peel on color, texture and oxidative stability of refrigerated ground pork patties submitted to high pressure processing. Innov. Food Sci. Emerg. Technol..

[6] Estévez M. (2011). Protein carbonyls in meat systems: A review. Meat Sci..

[7] Utrera M., Estévez M. (2012). Oxidation of myofibrillar proteins and impaired functionality: Underlying mechanisms of the carbonylation pathway. J. Agric. Food Chem..

[8] Estévez M., Luna C. (2017). Dietary protein oxidation: A silent threat to human health?. Crit. Rev. Food Sci. Nutr..

[9] Soladoye O.P., Juárez M.L., Aalhus J.L., Shand P., Estévez M. (2015). Protein oxidation in processed meat: Mechanisms and potential implications on human health. Compr. Rev. Food Sci. Food Saf..

[10] Viana F.M., Canto A.C.V.C.S., Costa-Lima B.R.C., Salim A.P.A.A., Conte-Junior C.A. (2017). Color stability and lipid oxidation of broiler breast meat from animals raised on organic versus non-organic production systems. Poult. Sci..

[11] Esterbauer H. (1993). Cytotoxicity and genotoxicity of lipid-oxidation products. Am. J. Clin. Nutr..

[12] Silva F.A.P., Estévez M., Ferreira V.C.S., Silva S.A., Lemos L.T.M., Ida E.I., Shimokomaki M., Madruga M. (2018). Protein and lipid oxidations in jerky chicken and consequences on sensory quality. LWT.

[13] Maloney J.F., Labuza T.P., Wallace D.H., Karel M. (1966). Autoxidation of methyl linoleate in freeze-dried model systems. I. Effect of water on the autocatalyzed oxidation. J. Food Sci..

[14] Labuza T.P. (1982). The effect of water activity on reaction kinetics of food deterioration. Food Technol..

[15] Estévez M., Padilla P., Carvalho L., Martín L., Carrapiso A., Delgado J. (2019). Malondialdehyde interferes with the formation and detection of primary carbonyls in oxidized proteins. Redox Biol..

[16] Hernández P., Navarro J.L., Toldra F. (1999). Lipolytic and oxidative changes in two Spanish pork loin products: Dry-cured loin and pickled-cured loin. Meat Sci..

[17] Estévez M., Morcuende D., Cava R. (2003). Oxidative and colour changes in meat from three lines of free-range reared Iberian pigs slaughtered at 90 kg live weight and from industrial pig during refrigerated storage. Meat Sci..

[18] Faustman C., Sun Q., Mancini R., Suman S.P. (2010). Myoglobin and lipid oxidation interactions: Mechanistic bases and control. Meat Sci..

[19] Pateiro M., Franco D., Carril J.A., Lorenzo J.M. (2015). Changes on physico-chemical properties, lipid oxidation and volatile compounds during the manufacture of celta dry-cured loin. J. Food Sci. Technol..

[20] Shikha Ojha K., Granato D., Rajuria G., Barba F.J., Kerry J.P., Tiwari B.K. (2018). Application of chemometrics to assess the influence of ultrasound frequency, *Lactobacillus sakei* culture and drying on beef jerky manufacture: Impact on amino acid profile, organic acids, texture and colour. Food Chem..

[21] Wang X., Zhang Y., Ren H., Zhan Y. (2018). Comparison of bacterial diversity profiles and microbial safety assessment of salami, Chinese dry-cured sausage and Chinese smoked-cured sausage by high-throughput sequencing. LWT.

[22] Yang Q., Sun D.W., Cheng W. (2017). Development of simplified models for nondestructive hyperspectral imaging monitoring of TVB-N contents in cured meat during drying process. J. Food Eng..

[23] Granato D., Putnik P., Kovačević D.B., Santos J.S., Calado V., Rocha R.S., Cruz A.G., Jarvis B., Rodionova O.Y., Pomerantsev A. (2018). Trends in chemometrics: Food authentication, microbiology, and effects of processing. Compr. Rev. Food Sci. Food Saf..

[24] Nunes C.A., Alvarenga V.O., Souza Sant’Ana A., Santos J.S., Granato D. (2015). The use of statistical software in food science and technology: Advantages, limitations and misuses. Food Res. Int..

[25] Incaper, Instituto Capixaba de Pesquisa, Assistência Técnica e Extensão Rural Monitoramento Agroclimático—Venda Nova do Imigrante/ES. https://meteorologia.incaper.es.gov.br/monitoramento-venda_nova_do_imigrante.

[26] Yin M.C., Faustman C., Riesen J.W., Williams S.N. (1993). α-Tocopherol and ascorbate delay oxymyoglobin and phospholipid oxidation in vitro. J. Food Sci..

[27] Joseph P., Suman S.P., Rentfrow G., Li S., Beach C.M. (2012). Proteomics of muscle-specific beef color stability. J. Agric. Food Chem..

[28] Oliver C.N., Ahn B.W., Moerman E.J., Goldstein S., Stadtman E.R. (1987). Age-related changes in oxidized proteins. J. Biol. Chem..

[29] Armenteros M., Heinonen M., Ollilainen V., Toldrá F., Estévez M. (2009). Analysis of protein carbonyls in meat products by using the DNPH-method, fluorescence spectroscopy and liquid chromatography–electrospray ionisation–mass spectrometry (LC–ESI–MS). Meat Sci..

[30] Canto A.C.V.C.S., Costa-Lima B.R.C., Suman S.P., Monteiro M.L.G., Viana F.M., Salim A.P.A.A., Nair M.N., Silva T.J.P., Conte-Junior C.A. (2016). Color attributes and oxidative stability of *Longissimus lumborum* and psoas major muscles from Nellore bulls. Meat Sci..

[31] Mercier Y., Gatellier P., Viau M., Remignon H., Renerre M. (1998). Effect of dietary fat and vitamin E on colour stability and on lipid and protein oxidation in Turkey meat during storage. Meat Sci..

[32] AMSA (2012). Meat Color Measurement Guidelines.

[33] Serra X., Ruiz-Ramírez J., Arnau J., Gou P. (2005). Texture parameters of dry-cured ham m. biceps femoris samples dried at different levels as a function of water activity and water content. Meat Sci..

[34] Harkouss R., Astruc T., Lebert A., Gatellier P., Loison O., Safa H., Portanguen S., Parafita E., Mirade P. (2015). Quantitative study of the relationships among proteolysis, lipid oxidation, structure and texture throughout the dry-cured ham process. Food Chem..

[35] Ruiz-Ramírez J., Serra X., Arnau J., Gou P. (2005). Profiles of water content, water activity and texture in crusted dry-cured loin and in non-crusted dry-cured loin. Meat Sci..

[36] Barden L., Decker E.A. (2016). Lipid oxidation in low-moisture food: A review. Crit. Rev. Food Sci. Nutr..

[37] Lund M.N., Heinonen M., Baron C.P., Estévez M. (2011). Protein oxidation in muscle foods: A review. Mol. Nutr. Food Res..

[38] Utrera M., Estévez M. (2013). Oxidative damage to poultry, pork, and beef during frozen storage through the analysis of novel protein oxidation markers. J. Agric. Food Chem..

[39] Shao J.H., Deng Y.M., Jia N., Li R.R., Cao J.X., Liu D.Y., Li J.R. (2016). Low-field NMR determination of water distribution in meat batters with NaCl and polyphosphate addition. Food Chem..

[40] Carneiro C.S., Mársico E.T., Ribeiro R.O.R., Conte-Júnior C.A., Mano S.B., Augusto C.J.C., Jesus E.F.O. (2016). Low-Field Nuclear Magnetic Resonance (LF NMR 1H) to assess the mobility of water during storage of salted fish (*Sardinella brasiliensis*). J. Food Eng..

[41] Böhner N., Hösl F., Rieblinger K., Danzl W. (2014). Effect of retail display illumination and headspace oxygen concentration on cured boiled sausages. Food Packag. Shelf Life.

[42] Estévez M., Ventanas S., Cava R. (2005). Protein oxidation in frankfurters with increasing levels of added rosemary essential oil: Effect on color and texture deterioration. J. Food Sci..

[43] Ganhão R., Morcuende D., Estévez M. (2010). Protein oxidation in emulsified cooked burger patties with added fruit extracts: Influence on colour and texture deterioration during chill storage. Meat Sci..

[44] Lobo F., Ventanas S., Morcuende D., Estévez M. (2016). Underlying chemical mechanisms of the contradictory effects of NaCl reduction on the redox-state of meat proteins in fermented sausages. LWT Food Sci. Technol..

[45] Fuentes V., Ventanas J., Morcuende D., Estévez M., Ventanas S. (2010). Lipid and protein oxidation and sensory properties of vacuum-packaged dry-cured ham subjected to high hydrostatic pressure. Meat Sci..

[46] Estévez M., Xiong Y. (2019). Intake of oxidized proteins and amino acids and causative oxidative stress and disease. J. Food Sci..

[47] Feeney R.E., Blankenhorn G., Dixon H.B. (1975). Carbonyl-amine reactions in protein chemistry. Advances in Protein Chemistry.

