# Supplementary material for: A Chemometric Approach to Establish Underlying Connections between Lipid and Protein Oxidation and Instrumental Color and Texture Characteristics in Brazilian Dry-cured Loin
Source: Foods. 2020 Apr 24;9(4):536. doi: 10.3390/foods9040536 (PMC7231182; doi:10.3390/foods9040536)
Supplement: Supplementary file 1 [file foods-09-00536-s001.pdf]

Table S1: Overall correlation between significative physicochemical characteristics (cluster analysis) on Brazilian dry-cured loin

	TBARS	Carbonyls	Hardness	Gumminess	Chewiness	a	b	Chroma	R(630/580)	Cured color
TBARS	1.00	0.50**	-0.51**	-0.55**	-0.48*	-0.70**	-0.47*	-0.61**	-0.65**	-0.69**
Carbonyls		1.00	-0.65*	-0.16	0.27	-0.48*	-0.49*	-0.51**	-0.33	-0.40*
Hardness			1.00	0.94**	0.78**	0.45*	0.26	0.37	0.23	0.31*
Gumminess				1.00	0.94**	0.40*	0.13	0.27	0.29	0.35*
Chewiness					1.00	0.33	0.01	0.17	0.32	0.35
a						1.00	0.80**	0.94**	0.83**	0.87**
b							1.00	0.95**	0.46*	0.50**
Chroma								1.00	0.67**	0.71**
R(630/580)									1.00	0.99**
Cured color										1.00

TBARS is expressed as mg malonaldehyde per kg of dry-cured meat and carbonyls is expressed as nmol of carbonyl per mg of protein. ** $p < 0.01$, * $p < 0.05$.
